# Dosimetric characteristics of LinaTech DMLC H multi leaf collimator: Monte Carlo simulation and experimental study

**DOI:** 10.1002/acm2.12055

**Published:** 2017-03-06

**Authors:** Mikaeil Molazadeh, Ahad Zeinali, Mostafa Robatjazi, Alireza Shirazi, Ghazale Geraily

**Affiliations:** ^1^ Department of Medical Physics and Biomedical Engineering Faculty of Medicine Tehran University of Medical Sciences Tehran Iran; ^2^ Department of Medical Physics Faculty of Medicine Urmia University of Medical Science Nazloo Campus Urmia Iran

**Keywords:** film dosimetry, IMRT QA, MLC transmission, Monte Carlo simulation, multi leaf collimator

## Abstract

This study evaluated the basic dosimetric characteristics of a Dynamic Multi Leaf Collimator (DMLC) using a diode detector and film measurements for Intensity Modulated Radiation Therapy Quality Assurance (IMRT QA). The EGSnrc Monte Carlo (MC) simulation system was used for the determination of MLC characteristics. Radiation transmission and abutting leaf leakage relevant to the LinaTech DMLC H were measured using an EDGE detector and EBT3 film. In this study, the BEAMnrc simulation code was used for modeling. The head of Siemens PRIMUS linac (6 MV) with external DMLC H was entered into a BEAMnrc Monte Carlo model using practical dosimetry data. Leaf material density, as well as interleaf and abutting air gaps were determined according to the computed and measured dose profiles. The IMRT QA field was used to evaluate the dose distribution of the simulated DMLC H. According to measurements taken with the EDGE detector and film, the total average measured leakage was 1.60 ± 0.03% and 1.57 ± 0.05%, respectively. For these measurements, abutting leaf transmission was 54.35 ± 1.85% and 53.08 ± 2.05%, respectively. To adapt the simulated leaf dose profiles with measurements, leaf material density, interleaf and abutting air gaps were adjusted to 18 g/cm^3^, 0.008 cm and 0.108 cm, respectively. Thus, the total average leakage was estimated to be about 1.59 ± 0.02%. The step‐and‐shoot IMRT was implemented and 94% agreement was achieved between the film and MC, using 3%‐3 mm gamma criteria. The results of this study showed that the dosimetric characteristics of DMLC H satisfied international standards.

## 1 Introduction

IMRT using photon beams is commonly performed with different types of Multi Leaf Collimators (MLCs). MLCs can be either integrated with the rest of the linac hardware, or added on externally. One of the primary goals of the QA in IMRT is to determine the dosimetric characterization of MLC.^1^ In IMRT treatments, in addition to the shielding of vital structures, MLCs are in charge of modulating the intensity of radiation beams, depending on the depth and type of tumor, and have many applications in the creation of volume dose distribution in the three‐dimensional form which is in accordance with the shape of the tumors. MLC provides all of these capabilities in IMRT treatments.

The specific features of any kind of MLC depend on the materials. Therefore, it is important to determine the dosimetric properties of the MLC and its effects on the dose distribution.^2^ Clinical consequences resulting from the incorrect determination of these features have been previously reported in IMRT treatments.^2^, ^3^, ^4^, ^5^ Studies considered leaf leakage shares as well as tongue and groove effects in dose calculations, especially in IMRT treatments with a long duration of radiation time which results in an increase in leaf transmission share in delivering an extra dose to the patient.^6^, ^7^ Therefore, an accurate determination of the dosimetric characteristics of MLCs using an appropriate dosimetry tool is one of the most important parameters in QA tests, in the field of IMRT treatments.^8^

Gafchromic films with special advantages and capabilities are among suitable tools recommended for IMRT QA.^9^, ^10^ The possibility of using it in water and in solid water phantoms, its high spatial resolution, the possibility of using it in a wide range of radiation and its low dependency on energy, makes the film a strong tool in the field of dosimetry.^11^ The BEAMnrc Monte Carlo code also has significant applications in MLC modeling^12^ and is widely used for accurate radiation dose calculations.

LinaTech Company has produced two types of DMLCs (DMLC H and DMLC M models). The DMLC H model has 102 leaves (51 pairs) while the DMLC M model has 54 leaves (27 pairs). These leaves can be embedded in any type of linac (Siemens, Varian, Elekta, etc.) and can also be used with any treatment planning software (Eclipse, Pinnacle, CMS, etc.). These external MLCs have the ability to implement both step‐and‐shoot and dynamic IMRT techniques.

In this study, several dosimetric properties of the DMLC H multi leaf collimator were measured and evaluated. In addition, to determine certain special characteristics of MLC, Monte Carlo modeling was considered.

## 2 Materials and methods

### 2.A Monte Carlo modeling

In accordance with the manufacturer's geometry and materials, a 6 MV medical linear accelerator (Siemens PRIMUS model) was modeled using the BEAMnrc/EGSnrc (Version V4‐r2‐4‐0) simulation software. All parts of the linac head including the target, primary collimator and flattening filter, monitor ion chamber, mirror and X‐Y jaws (secondary collimators) were modeled using modules provided by the code. The VARMLC module was used to simulate the external DMLC H. Using the DOSXYZnrc software, dose calculation was performed in a water phantom.

To reduce the simulation run time and increase the efficiency, variance reduction techniques were used in the simulations. The global cut‐off energy for electron and photon particles was set to 0.7 and 0.01 MeV, respectively. To increase the number of photons generated in the target, Directional Bremsstrahlung Splitting (DBS) was used.^13^ Therefore, to maximize dose and fluence efficiency at 6 MV beam energy, NBRSPL (DBS splitting number) was set to 1000. The DBS splitting field radius was equal to the side of the square field to be defined (square field defined at a certain distance from the target). Electron range rejection was also used with the ESAVE parameter, which is the energy threshold required to turn on the range rejection, set to 2 MeV.^13^, ^14^

The Monte Carlo simulations were validated in two steps. In the first step, the Siemens linear accelerator head (in the absence of MLC) was simulated and validated according to the practical measurements. In the second step, MLCs were added and validated according to the dosimetry data.

#### 2.A.1 Simulation of the Siemens PRIMUS linac

Realistic and reliable results are obtained when accurate details based on the manufacturer's data, are used for the simulation. One of the most important parameters in the accurate modeling of the linac is the target of the accelerator.^15^ Another important parameter is the simulation of the flattening filter, which is located inside the primary collimator and is used to flatten the beam at a certain depth. The average energy of the produced beam depends on the geometry and the materials used in this piece.^16^ Hence, the target and flattening filter play a significant role in MC simulation results; therefore, the target equipped with a flattening filter can be named the heart of the simulation.

**Figure 1:**
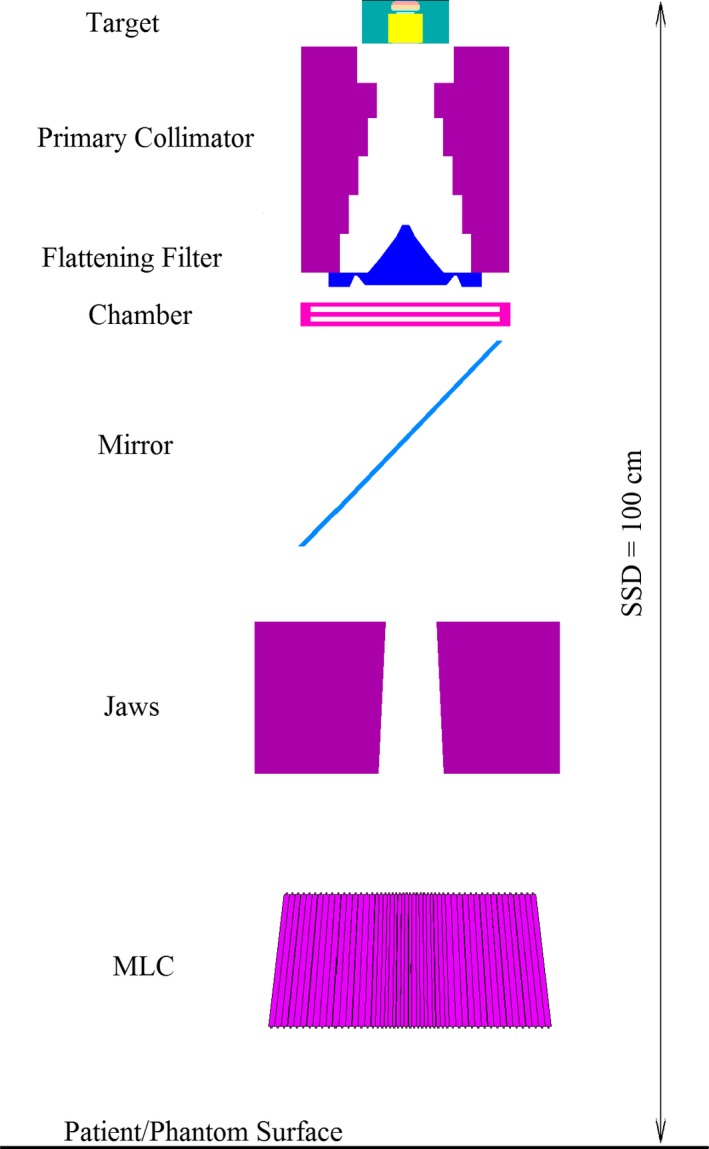
Graphical view of the simulated Siemens linear accelerator head (6 MV Primus) with LinaTech external DMLC H.

Other constituent parts of the head, including the parallel plate ionization chamber and mirror were simulated. Thereafter, two pairs of tungsten jaws which lie in a perpendicular direction to each other were modeled. These jaws were used for radiation beam collimation in the required field sizes. The geometric shape of the simulated Siemens linac head is shown in Fig. 1.

Source number 19 in BEAMnrc Monte Carlo code was used for modeling initial electron beam energy. This source has a monoenergetic beam with two‐dimensional distribution of Gaussian intensity.^17^ The initial electron beam parameters (energy and radius) were determined in accordance with the method proposed by Sheikh‐Bagheri and Rogers.^18^ According to this method, the energy and size of the electron beam will be determined if a good agreement is found between the simulated and measured data.^19^, ^20^ Electron beam energy in the range of 5.8–6.6 MeV and its Full Width at Half Maximum (FWHM) in the range of 0.8–2.2 mm was investigated in steps 0.1 MeV and 0.2 mm, respectively. By reviewing MC results with the Percentage Depth Dose (PDD) curves (for determining energy) and lateral dose profiles (for determining energy and especially FWHM), electron beam energy and its radius size were determined. These assessments were performed at different square field sizes and at various depths (d_max_, 5 and 10 cm).

Phase space files were used in the validation process. Phase space files with different arrangements of energies and FWHMs were generated at a 100 cm Source Surface Distance (SSD). Thereafter, symmetrical field sizes of 3, 5, 10, 15, and 30 cm^2^ were defined by the jaws. The number of histories in different field sizes was different. The number of electron particles irradiated to the target in the standard size field (10 × 10 cm^2^) was about 2 × 10^8^. For field sizes greater than 10 × 10 cm^2^, about 3 × 10^8^ electron particles were irradiated.

The generated phase space files in the linac isocenter were used as input data in the DOSXYZnrc code. In field sizes of 3 × 3 cm^2^ and 5 × 5 cm^2^, the surface of the water phantom was irradiated with 10 × 10^9^ particles. The particles are photons, electrons, and positrons but the primary particles are mainly photons. In the standard field size, the number of histories was set to 20 × 10^9^ and the number of photon particles increased with increase in field size. The global cut‐off energy for electron and photon particles was set to 0.7 and 0.01 MeV, respectively. NBRSPL was set to 1000. Depending on the field size, different computational resolutions were considered. Computational resolution in low‐dose gradient areas (the region of 80% profile relative to the central axis) and in regions with high‐dose gradient (penumbra regions), was set to 2 and 1 mm, respectively. In the direction of the central axis (CAX), the resolution was 1 mm. Therefore, five kinds of phantoms were defined and utilized in the five radiation fields. Considering the number of photon particles used in the simulation, the Monte Carlo uncertainty was less than 1% (2 SD).

The configuration and constituent materials of DMLC H were modeled based on the manufacturer's data; and the other simulation parameters relevant to DMLC H, including MLC density, Z focus of the leaf sides, Z_min_ (Z of the top of the MLC), interleaf air gap and abutting leaf gap were investigated. Since the average IMRT beam is about 10 × 10 cm^2^, this field size was selected to determine MLC leakage and transmission. The standard field size was defined using jaws while the MLC was removed from the radiation field (the MLC field size was 30 × 30 cm^2^). This field size was defined as the Reference Field Size (RFS). Under these conditions, another field called the Closed MLC (C‐MLC) was modeled in which MLCs were closed in the center (x = 0). Also, another field called Blocked MLC (B‐MLC) was designed. In B‐MLC, leaves on one side of the secondary collimators were closed. With the conditions mentioned above and by changing parameters related to the MLC, such as density, Z focus, Z_min_, abutting and interleaf air gaps, the dosimetric specifications of DMLC H were investigated.

#### 2.A.2 Simulation of the LinaTech DMLC H

**Figure 2:**
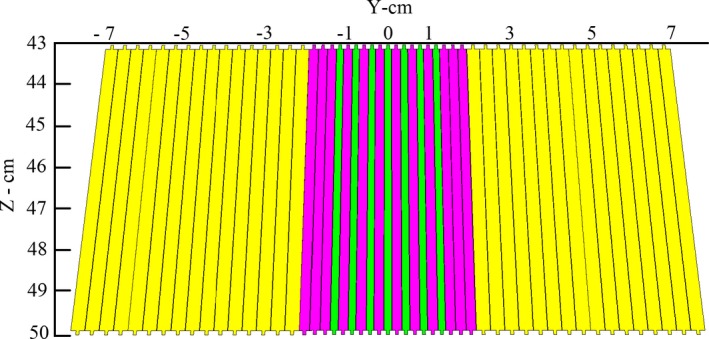
Arrangement of the DMLC H leaves in YZ plane. Seven pairs of leaves with thin width (green) and also 12 pairs of leaves (magenta) are located in the center of the MLC leaf bank. The remaining 32 pairs of leaves, from 51 leaf pairs, make up the outer part of the DMLC H.

DMLC H contains three leaf banks with different widths. The inner leaf bank is composed of seven leaf pairs with a width of 3.6 mm and 12 leaf pairs with a width of 4.8 mm. As shown in Fig. 2, internal leaves (19 pairs) are specially arranged beside each other. The external leaf bank is composed of 32 leaf pairs with a width of 6.9 mm. The positions of the leaves are projected on the isocenter plane. The MLC leaves are placed next to each other in the form of a convex and angled in a way that the divergence of radiation beams are taken into consideration (see Fig. 2). The Z focus parameter shows the leaf tip curve radius along the X axis in the direction of the radiation beam. The leaf tip along the Y axis does not have any curve. The maximum useful field size covered with this kind of MLC is about 30 × 30 cm^2^ in the isocenter area, and the MLC leaf length in the above mentioned area is 27.4 cm. These leaves have the capability of movement along the X axis and to deliver dose to the tumor, it can be arranged into an irregular shape. Screws at the top and bottom of the leaf have a width less than 1 mm, and their height is 1 mm. The height of MLC is about 7 cm. The upper edge of MLC is located at a distance of 47 cm from the linac target. In simulations, leaf ends were considered in a circular form with a curvature radius of 13 cm. For ease in the movement of adjacent leaves and to eliminate the friction caused by movement of adjacent leaves (adjacent leaf pairs), a small air gap called interleaf air gap was considered in the MLC design. Also, to prevent damage to leaves located opposite each other (abutting leaf pairs), there is another air gap called the abutting leaf gap. In fact, the leaves were separated by two types of air gaps which can be moved and controlled independently by the user. With regard to the properties and certain specifications mentioned about this type of MLC, among the existing modules in the code of BEAMnrc, the VARMLC component module was used for DMLC H simulation.

##### Interleaf leakage, intraleaf transmission and MLC leakage

Different simulation parameters, such as MLC material density, Z focus, interleaf air gap, Z_min_, and abutting leaf gap were chosen according to the measurements. By so doing, the difference between simulation and measurement results were obtained. The first three parameters were determined using the conditions created in the B‐MLC mode.

The inline profile diagram of the MLC leakage was simulated by applying different numerical changes in the three parameters (density, Z focus, and interleaf air gap). Thereafter, the results were compared with the leakage profiles obtained from the Radiochromic film and diode detector. For this purpose, MLC material density in the range of 16–19 g/cm^3^, Z focus in the range of −50 to +50 cm and air gap in the range of 0.004–0.03 cm were changed. First, to determine the air gap between adjacent leaves, the air gap value was set as 0.004 cm and then gradually increased. By adjusting the interleaf air gap in the mentioned range, the peaks and valleys on the inline leakage profile were observed. Then, by adjusting the density and Z focus, the best agreement between simulated and experimental dose profiles were obtained.

In BEAMnrc simulation, approximately 4 × 10^8^ particles were used for the B‐MLC field, of which about 31 × 10^6^ particles were registered in the phase space file located in the linac isocenter. Considering the number of photons irradiated to the surface of the phantom (4 × 10^9^ particles), the statistical uncertainty for the DOSXYZnrc calculations was less than 2% (1 SD). To extract the inline leakage profile, dose values in the B‐MLC field were normalized to the CAX dose value of the RFS field.

##### Abutting air gap

The abutting air gap parameter was extracted through the C‐MLC field. To determine the abutting leaf gap, a simulation was conducted according to the C‐MLC field. Several simulations were performed with an air gap range of 0.008–0.2 cm. About 4 × 10^8^ particles were run in BEAMnrc for simulating C‐MLC and the number of particles written in the phase space file was about 31 × 10^6^ particles. In DOSXYZnrc simulation, the number of particles in the C‐MLC test was 4 × 10^9^ particles. To extract the cross‐line profile, computed dose values in the C‐MLC field were normalized to the CAX dose value of the RFS field.

##### Tongue and groove design

The irregular MLC pattern was designed for investigating the tongue and groove design. The central leaf pairs (leaf pair 26) against each other at a distance of −1 cm from the central axis of the beam were closed. Other leaves on both sides of the central leaf were alternately opened and closed. Opened leaves were placed outside the radiation field. This kind of leaf arrangement was named Alternated MLC pattern (A‐MLC). The Z_min_ parameter was obtained using the A‐MLC pattern. A 10 × 10 cm^2^ jaw‐defined field was used in this test. Measurements were made using a silicon diode detector in water and film in a solid water phantom (at 5 cm depth and 100 cm SSD). The lateral dose profiles obtained from the EBT3 film, EDGE detector and MC simulation were normalized to 100 (to their maximum relative dose). In MC simulation, the Z_min_ parameter was changed from 41 to 45 cm with intervals of 0.5 cm. In the BEAMnrc simulation, 25 × 10^7^ particles were used for the A‐MLC field. Also, in the DOSXYZnrc simulation, the number of histories in the A‐MLC field was set as 3 × 10^9^ particles.

In all of the experimental measurements and Monte Carlo calculations, the SSD was set at 100 cm while the depth was set at 5 cm. The A‐MLC, B‐MLC, and C‐MLC fields were measured under similar experimental conditions in terms of depth and SSD using an EDGE detector in water and EBT3 film in a solid water phantom. Subsequently, to validate the simulated DMLC H, several square fields (opened by MLC) were assessed with the practical measurements.

### 2.B Experimental measurements

#### 2.B.1 Dosimetry via detectors (Semiflex and EDGE)

**Figure 3:**
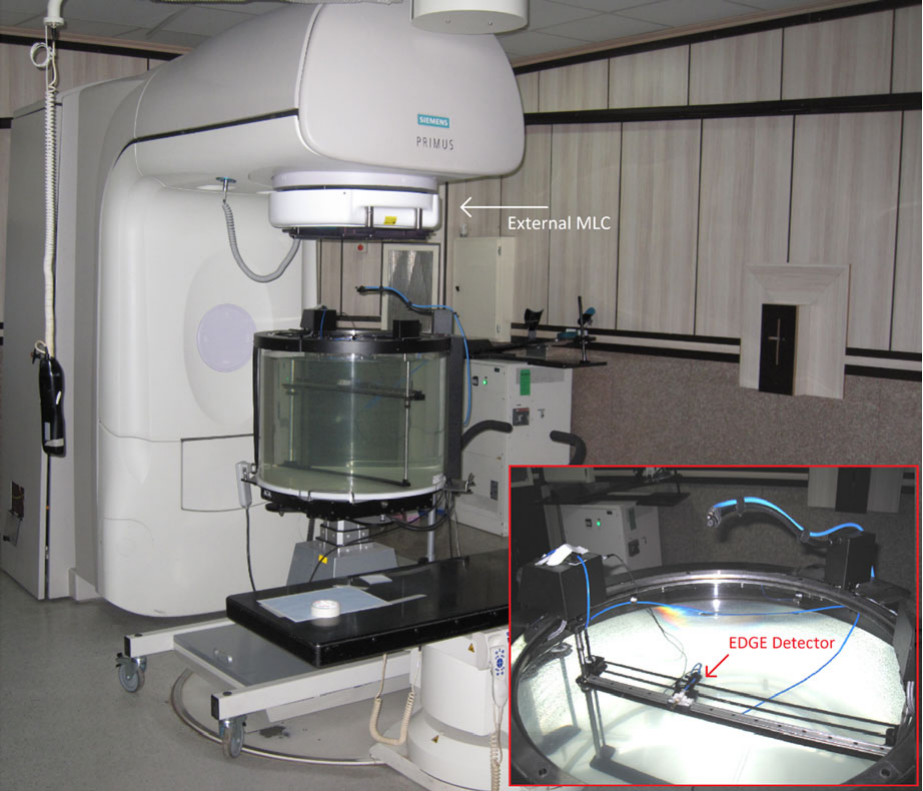
Equipment used for practical dosimeters, which consist of the SNC 3D Scanner, SNC EDGE detector and the PTW Semiflex ionization chamber.

To evaluate the results of MC simulation with experimental measurements, practical dosimetry was performed according to the IAEA TRS 398 protocol in water,^21^ and the data was collected using a Semiflex cylindrical ionization chamber with 0.125 cm^3^ nominal sensitive volume (type TN31010, PTW‐Freiburg, Germany) in a motorized 3D Scanner™ (model 1230, Sun nuclear Corporation, Florida, USA). The SNC Dosimetry Software (version 1.3.2) was used to control the motor system in a 3D Scanner. Moreover, this software was used for data collection and processing. In the SNC 3D Scanner, after the detectors were attached to the scanning system, the water tank which uses water sensors at three different locations in the tank was automatically leveled. Thereafter, using auto‐setup procedure, the position of the central axis point was automatically (without manual setup) determined. In addition, the Sun Nuclear EDGE Detector^TM^ (model 1118) was used for measurements related to A‐MLC, B‐MLC, C‐MLC, and radiation fields smaller than 5 × 5 cm^2^. The EDGE detector is a kind of silicon diode detector with an active volume of 0.000019 cm^3^. Unlike the Semiflex detector, the EDGE detector is oriented horizontally so that the top surface which is labeled with a crosshair sign perpendicular to the central axis of the beam and toward the source is set. After positioning the effective point of measurement of the EDGE detector (about 0.5 mm below the surface of the water), the probe moves to the desired depth and scans are taken. The Siemens PRIMUS accelerator and external MLC mounted on the linac head with the above‐mentioned equipment are shown in Fig. 3.

#### 2.B.2 EBT3 film dosimetry

Gafchromic EBT3 films (lot #: 04201502, 8 × 10 inch sheets) were used in practical dosimetry experiments. Practical measurements using the EBT3 film were performed in a solid water phantom and in the measurements, film dosimetry protocols (AAPM Task Group Report 55)^22^ and technical considerations as recommended by vendors,^23^ were considered. Films were scanned using a Microtek 9800XL flatbed scanner in 48‐bit RGB (Red‐Green‐Blue color representation) format and analyzed using MATLAB software (R2015a 8.5). The red channel of the EBT3 film was used in film dosimetry.

Regarding the fact that the least area of the film should be 25 cm^2^,^23^ film pieces of 5 × 5 cm^2^ which were cut from the same sheet were used to obtain calibration curves. For the identification and separation of film pieces with similar dimensions from each other and to determine shorter length than the original sheet of the film, all the pieces were numbered by writing numbers on the top left hand corner. Films were placed at a depth of 5 cm away from the solid water phantom, at the SSD of 100 cm. A field size of 10 × 10 cm^2^ was set by jaws. According to the stated conditions, films with 20 different dose levels, including 0, 30, 50, 100, 150, 200, 250, 300, 350, 400, 450, 500, 700, 850, 1000, 1200, 1400, 1600, 1800, and 2000 cGy were irradiated. Also, to provide full backscatter condition, the total thickness of the solid water phantom below the film was about 15 cm.^24^

The EBT3 films were scanned 48 h after irradiation using the Microtek scan wizard pro V7.26 software. To improve film response and reduce the error (about 9%) caused by the incorrect placement of the film on the scanner, it was scanned in the landscape orientation so that the shorter side of the film was placed along the long side of the scanner.^25^ To obtain raw data, the use of any type of filter and image processing tools was avoided. The films were scanned in the full dynamic range condition, in the transmission mode and in 48‐bit RGB color mode with a spatial resolution of 127 dpi (0.2 mm) and saved in the TIFF file format.

For the extraction of calibration curve, pieces of film were exposed to doses ranging from 0 to 2000 cGy. To reduce the film dose–response uncertainty and improve the accuracy of the sensitometric calibration curve, each dose level was repeated three times and the mean net Optical Density (netOD) was used to obtain the calibration curve. In order to deliver precise doses to the films, the value of the absorbed dose corresponding to each dose level was obtained with a PTW Semiflex chamber of 0.125 cm^3^ (model 31010) mounted at a depth of 5 cm within a solid water phantom. All measurements were performed according to the IAEA TRS 398 protocol.^21^

To obtain netOD, prior to exposure, the initial OD (OD_initial_) of the unirradiated films was calculated using eq. (1).

(1) $$OD_{initial}=-log_{10}\frac{\left(PV_{unexp}-PV_{unopacue}\right)}{\left(PV_{unblank}-PV_{unopaque}\right)}$$

where PV_unexp_, PV_unopaque_ and PV_unblank_ represent the pixel values of the unexposed film, opaque sheet scan and pixel value of the blank screen, respectively. Finally, after irradiation, the netOD was obtained using eq. (2).

(2) $$netOD=-log_{10}\frac{\left(PV_{exp}-PV_{opacue}\right)}{\left(PV_{blank}-PV_{opaque}\right)}-OD_{initial}$$

where PV_exp_, PV_opaque_, and PV_blank_ represent the pixel values of the exposed film, opaque sheet scan and pixel value of the blank screen, respectively. It should be noted that to obtain the netOD, the OD_initial_ of each piece of film was calculated separately. In fact, a generic background was not used. Instead, the OD_initial_ was used as the background.

The experimental dose, fitting dose and the total dose uncertainties were estimated by error propagation as proposed by Devic et al.^24^ For the analysis of films, an area of 1 cm^2^ was selected from the central part of the film (50 × 50 pixels).^24^ The Levenberg–Marquardt algorithm was used to obtain an appropriate calibration curve and minimize the fitting uncertainty.^26^, ^27^ Finally, the calibration curve was obtained by fitting a third‐degree polynomial curve.

In A‐MLC, B‐MLC, C‐MLC, and RFS fields, films were cut into 4 × 25 cm^2^ and then in accordance with the intended position, they were placed in a solid water phantom. For B‐MLC 11500 MU (Monitor Unit), C‐MLC 1000 MU, A‐MLC, and RFS field 300 MU were exposed to films. For square fields (with and without MLC), the films were irradiated with 300 MU. Also, the required MU for measuring MLC leakage (in B‐MLC field) using the EDGE detector was less than 1000.

All radiations were carried out by Siemens Primus linac (6 MV) and before irradiation; output was tuned to 1 cGy/MU. The spatial resolution of MC calculations and detector readings was 1 mm.

### 2.C IMRT QA field

The QA tests are one of the basic tests required to commission different computing systems in radiotherapy centers.^5^ One of the tests recommended by the American Association of Physicists in Medicine (AAPM) is test No. 4 of AAPM TG‐119.^28^ According to this test, the “C” shaped PTV has a length of 8 cm, the inner radius of 1.5 cm, and outer radius of 3.7 cm. A cylindrical organ at risk is located inside the target and the center is concentric with the center of the PTV. In this study, to assess the dose distribution calculations done by MC simulations, the above test was modeled as segmental‐IMRT using DMLC H in the step‐and‐shoot technique. The obtained results were compared with the EBT3 film dose distribution. The IMRT treatment planning was performed with the TiGRT V7.2.24 treatment planning system (LinaTech Co., Sunnyvale, CA, USA). In treatment planning, 35 segments were designed at nine fields with gantry angle intervals of 40°. In addition, the accuracy of the dose distribution results obtained from TiGRT was compared with the MC simulated results.

In all the assessments, an area of 10 × 10 cm^2^ (with a pixel spacing of 1 mm) was selected, and thus, approximately 10000 pixels were analyzed using the gamma index. Gamma analysis is a dimensionless function that simultaneously takes both Dose Difference (DD) and Distance‐To‐Agreement (DTA) criteria into account.^29^ For gamma analysis, the dose distribution obtained from the Monte Carlo and EBT3 film via MATLAB software was converted to the DICOM‐RT DOSE format and analyzed by the γ‐index dosimetry module of the VeriSoft/MEPHYSTO software (version 5.1, PTW, Freiburg, Germany). Through the gamma analysis option,^29^ the dose distributions were evaluated quantitatively and graphically. In quantitative studies, film measurements were chosen as the reference dose distribution and the other dose distributions were compared against it.

## 3 Results

### 3.A Experimental validation of Monte Carlo modeling

**Figure 4:**
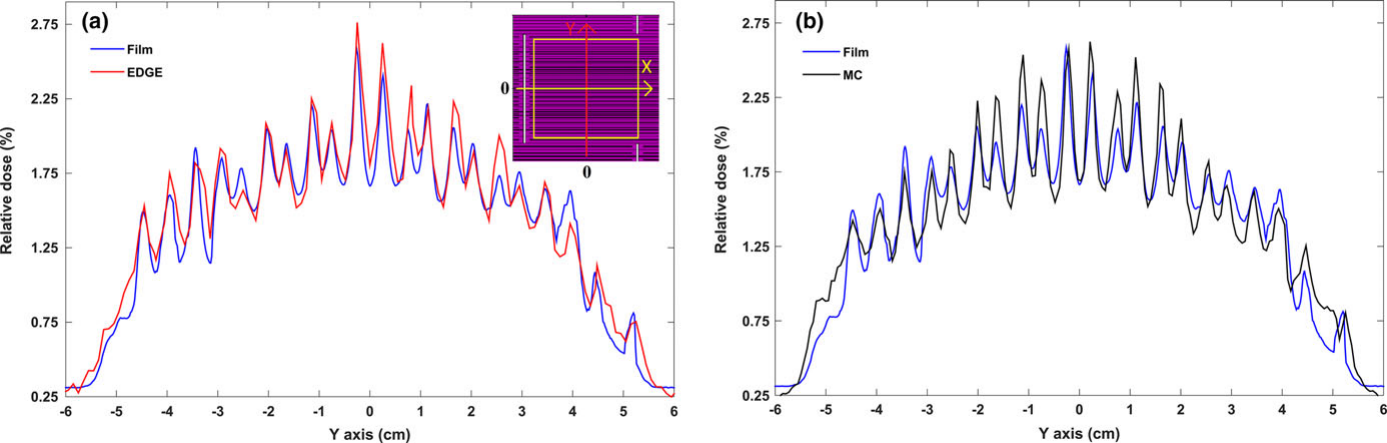
Validation of the MC modeling of the linac head (without MLC) using practical dosimetry: (a) percentage depth dose profiles of the 3 × 3, 5 × 5, 10 × 10, 15 × 15, and 30 × 30 cm^2^ field size; (b) lateral dose distribution profiles for the same field sizes at a depth of 5 cm. The maximum dose in the current dose distribution profiles was normalized to 60, 70, 80 90 and 100, respectively. Continuous blue lines are the measurements of the EBT3 Gafchromic film whereas the red and yellow squares indicate the results of MC simulation.

**Figure 5:**
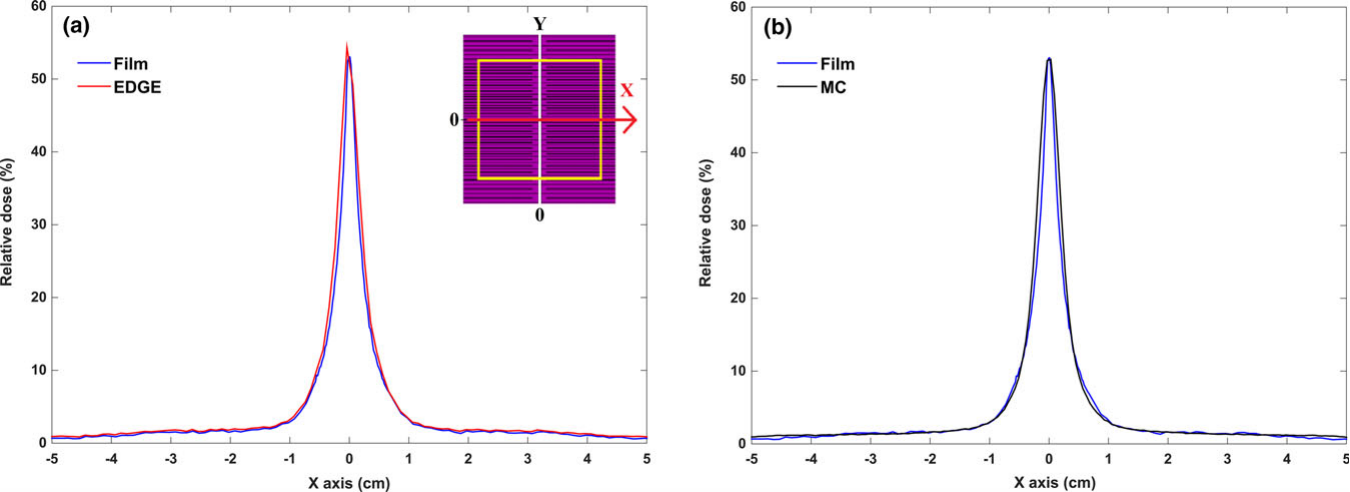
Comparison of the dose distribution curves along the central axis of the beam (a) and perpendicular to the central axis of the beam (b) of the external DMLC H. The square fields of 3, 5, 10, 15 and 30 × 30 cm^2^ normalized to 60, 70, 80, 90, and 100, respectively. The continuous lines (blue) correspond to the EDGE detector readouts and the squares (red and yellow) correspond to the MC simulation.

The results of this study showed a good agreement between the simulated and experimental data when the combination of 6.2 MeV and 0.09 mm for the incident electron beam parameters were considered. In the build‐up area, discrepancies of about 4% were observed between PDD curves of the MC simulation and measurements, and this discrepancy was estimated to be less than 2% in the area after the maximum dose. In the flat area of the lateral dose profiles, the amount of DD between MC results and practical measurements was about 2% and for all field sizes, the value of DTA in areas with high‐dose gradient was less than 1 mm. PDD and profile curves related to MC simulation (jaw‐defined open fields) and practical measurements are shown in Fig. 4. Beam profiles and PDD curves (defined by MLC) relevant to the EDGE detector and MC simulation for the 6 MV photon beam at a depth of 5 cm are shown in Fig. 5. The most acceptable agreements for density with a value of 18 g/cm^3^, for Z focus equals −10 cm while the interleaf air gap equals 0.008 cm. Table 1 shows a summary of the results of changes in various parameters.

**Table 1:** Summary of the MC simulation results as compared to the results of film dosimetry to determine the characteristics of the DMLC H by changing various parameters (density, interleaf air gap and Z focus)

No.	Material Density (g/cm^3^)	Interleaf air gap (cm)	Z focus (cm)	Mean interleaf leakage (mean ± SD) (Δ)	Mean intraleaf transmission (mean ± SD) (Δ)	Total average leakage (mean ± SD) (Δ)
1	17.8	0.008	−20	2.03 ± 0.09% (9.73)	1.43 ± 0.03% (5.15)	1.64 ± 0.01% (4.46)
2	18	0.008	10	1.74 ± 0.02% (−5.95)	1.32 ± 0.08% (−2.94)	1.50 ± 0.05% (−4.46)
3	18	0.008	0	2.61 ± 0.08% (41.08)	1.60 ± 0.06% (17.65)	1.99 ± 0.10% (26.75)
4	18	0.007	−10	1.77 ± 0.06% (−4.32)	1.22 ± 0.01% (−10.29)	1.54 ± 0.03% (−1.91)
5	18	0.008	−10	1.95 ± 0.04% (5.41)	1.32 ± 0.05% (−2.94)	1.59 ± 0.02% (1.27)
6	18	0.009	−10	1.99 ± 0.03% (7.57)	1.39 ± 0.06% (2.21)	1.63 ± 0.01% (3.82)
7	18.2	0.008	−10	1.75 ± 0.09% (−5.41)	1.25 ± 0.03% (−8.09)	1.55 ± 0.08% (−1.27)

Δ= % Difference compared with the results of film.

### 3.B Dosimetric characteristics of the DMLC H

#### 3.B.1 Leakage parameters

**Figure 6:**
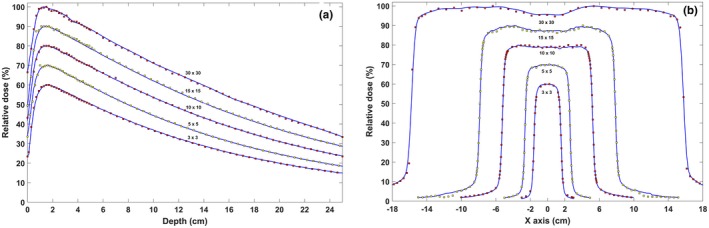
(a) Leakage profile of EBT3 film and comparison with the transmission profile as measured using the EDGE detector and (b) leakage dose distribution predicted by BEAMnrc. MLC leakage profiles were obtained along the Y axis (as shown in the left diagram placed in the upper right corner). The results normalized to standard open field (10 × 10 cm^2^). According to Monte Carlo calculations in B‐MLC field, leaf density and interleaf air gap values were estimated to be 18 g/cm^3^ and 0.008 cm, respectively.

**Figure 7:**
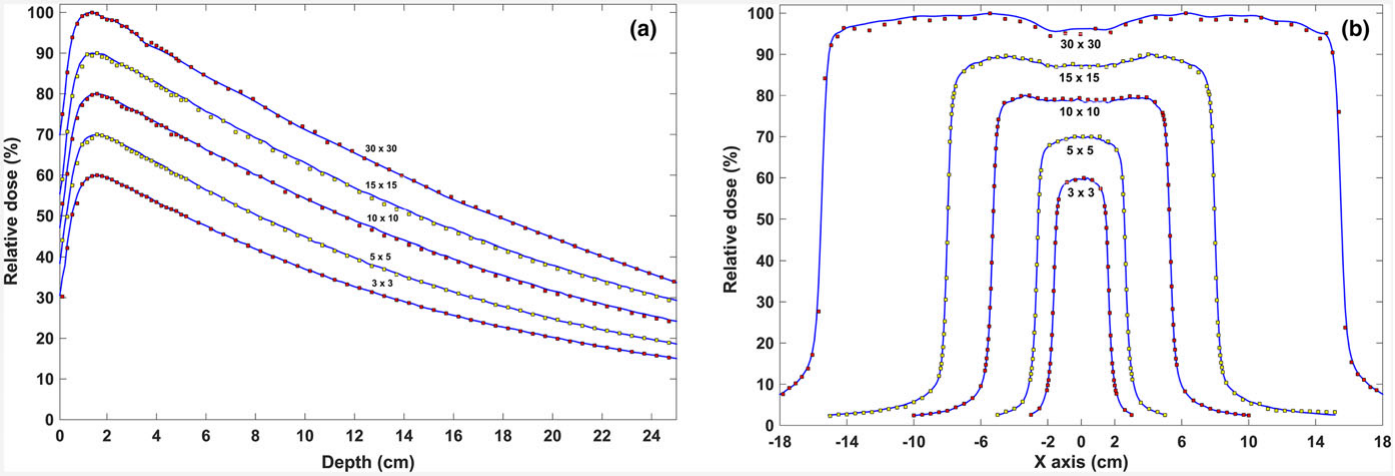
(a) Determination of the amount of leakage caused by the abutting air gap between the leaves via film dosimetry and diode detector measurements. (b) Monte Carlo calculations indicate a 52.80% leakage by taking 0.108 cm air gap between the leaves. According to the left graph in the upper right corner, dose distribution of the abutting air gap was obtained along the X axis and all results were normalized to the opened standard field. Yellow square indicates the field size is 10 × 10 cm^2^.

According to measurements carried out using the EDGE detector and EBT3 film, the total average measured leakage was 1.60 ± 0.03% and 1.57 ± 0.05%, respectively. The average value of abutting leaf leakage obtained using a diode detector and film was 54.35 ± 1.85% and 53.08 ± 2.05%, respectively. Figures 6(a) and 7(a) present the corresponding dose profiles. There is a good agreement between the film and the diode detector data. Although the maximum discrepancy between the dose valleys and peaks was 15.39%, the average difference between the EBT3 film and EDGE detector for leakage was estimated to be about 1.91%. For the EDGE detector, the average interleaf leakage was 1.89 ± 0.11% and the average intraleaf transmission was 1.37 ± 0.08%. In film dosimetry, these parameters were 1.85 ± 0.05% and 1.36 ± 0.09%, respectively. It should be noted that the uncertainty in film dosimetry was less than 5%.

The leakage dose distribution of the DMLC H related to MC, EDGE, and EBT3 film are shown in Figs. 6(a) and 6(b). According to the parameters that were determined (density = 18 g/cm^3^, Z focus = −10 cm and interleaf air gap = 0.008 cm), the average values of total leakage, interleaf leakage and intraleaf transmission were 1.59 ± 0.02%, 1.95 ± 0.04%, and 1.32 ± 0.05%, respectively. The maximum discrepancy between the peaks and valleys of the MC simulation and EDGE detector was 17.38%. This difference between the film and MC simulation was 15.88%.

#### 3.B.2 Abutting air gap

In determining abutting air gap, a good agreement was observed with 0.108 cm. Thus, based on the Monte Carlo simulation, the amount of abutting leaf leakage was 52.80 ± 0.06%. According to these results, the leakage discrepancy for abutting air gap between the MC simulation and EDGE detector was −2.85% and between the MC simulation and EBT3 film, it was −0.53%. It is evident that the presence of abutting air gap causes an increase in approximately 53% more than the prescribed dose. Figures 7(a) and 7(b) show the abutting leaf dose profile obtained from the MC simulation, diode detector and film measurements in the direction of the X axis.

To better analyze the results, a summary of the dosimetric parameters of DMLC H are presented in Table 2.

**Table 2:** Dosimetric characteristics of LinaTech DMLC H multi leaf collimator. The EDGE and Monte Carlo results were compared with the film results

	Mean leakage (mean ± SD)	Interleaf leakage (mean ± SD)	Intraleaf transmission (mean ± SD)	Abutting air gap leakage (mean ± SD)
EBT3 Film	1.57 ± 0.05%	1.85 ± 0.05%	1.36 ± 0.09%	53.08 ± 2.05%
EDGE	1.60 ± 0.03% (Δ = 1.91)	1.89 ± 0.11% (Δ = 2.16)	1.37 ± 0.08% (Δ = 0.74)	54.35 ± 1.85% (Δ = 2.39)
Monte Carlo	1.59 ± 0.02% (Δ = 1.27)	1.95 ± 0.04% (Δ = 5.41)	1.32 ± 0.05% (Δ = −2.94)	52.80 ± 0.06% (Δ = −0.53)

Δ= % Difference compared with the results of film.

#### 3.B.3 Tongue and groove design

**Figure 8:**
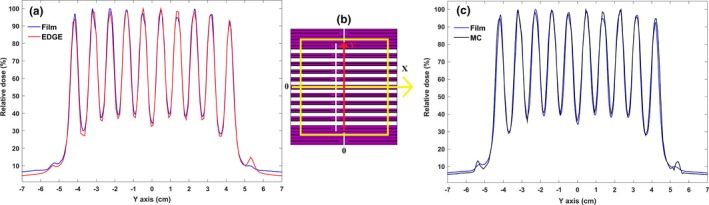
Assessment of the A‐MLC pattern profile related to tongue and groove design: (a) The profiles relating to this pattern using film and diode detector measurements; (b) Tongue and groove design. Yellow square indicates the field size is 10 × 10 cm^2^. In this MLC pattern position of the two leaves closed in the field at a distance of −1 cm from the central axis of the beam (−1 cm off‐axis from CAX) and location of the two leaves opened are outside the radiation field; (c) Dose distribution of the tongue and groove pattern that is derived from MC calculations. The results were normalized to 100 (normalized to the maximum dose value multiplied by 100).

The impact of the tongue and groove design in DMLC H modeling was assessed. The dose profiles associated with this test are illustrated in Figs. 8(a) and 8(c). By choosing Z_min_ equal to 44 cm, MC dose calculations demonstrated a good agreement with the measurements. The outcome of this study showed that the average discrepancy between the MC simulation and diode detector is 6.28 ± 0.38% and between the MC simulation and film is 4.77 ± 0.23%.

### 3.C IMRT QA field

**Figure 9:**
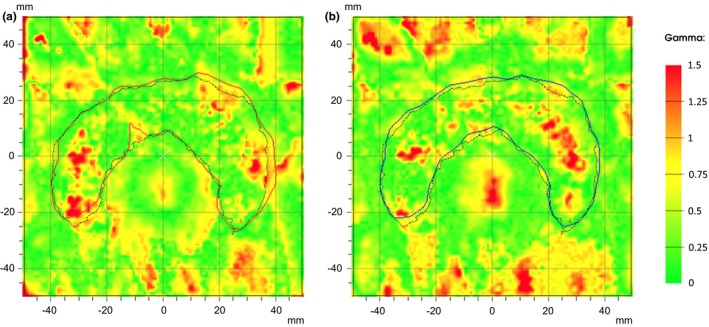
(a) Gamma dose distribution (with 3%‐3 mm gamma criteria) related to EBT3 film and MC. The red continuous line corresponds to the 100% isodose line of MC and dashed line corresponds to the film at the same isodose. (b) 2D gamma map of EBT3 film and TiGRT treatment planning system with 3%‐3 mm gamma criteria. 100% isodose line of the TiGRT was visualized by the blue continuous line, whereas this isodose line for EBT3 film is black‐dashed line.

The planar dose distributions between the planned and actual dose distributions were assessed using gamma function. The gamma test results for different criteria are provided in Table 3. Figure 9(a) shows the gamma dose map between MC and the film with 3%‐3 mm gamma criteria. The results of the gamma analysis using 3%‐3 mm, 4%‐4 mm and 5%‐4 mm criteria showed that the agreement between MC and the film are 94, 98.7, and 99.5%, respectively. Figure 9(b) shows the quantitative dose distribution for a “C” shaped MLC field between TiGRT's Full Scatter Convolution (FSC) algorithm and EBT3 film with 3%‐3 mm gamma criteria. According to the above‐mentioned gamma indices, 92.1, 98, and 99.1% of the pixels passed the gamma analysis, respectively.

**Table 3:** Comparison of pass rates for Monte Carlo simulation and FSC algorithm with various sets of gamma criteria. Two‐dimensional dose distribution of the EBT3 film was selected as the reference dose distribution

	% Fraction of pixels satisfying gamma criteria				
DD‐DTA	3%‐3 mm	4%‐3 mm	5%‐3 mm	4%‐4 mm	5%‐4 mm
Monte Carlo	94	97.6	98.5	98.7	99.5
FSC	92.1	95.8	97.9	98	99.1

## 4 Discussion

In this study, the dosimetric properties of the LinaTech DMLC H were determined using experimental measurements and Monte Carlo simulations. Moreover, the simulated and computed dose distributions were evaluated as compared to the measured dose distribution in the complex IMRT plan. The compliance of the measured dose distribution (film) with the calculated dose distributions (MC and FSC) requires accurate determination of the dosimetric parameters of the MLC by an ideal detector. Several studies have evaluated clinical effects related to changes in the dosimetric properties of MLC.^3^, ^4^, ^30^ Whenever the accuracy of the instrument used in determining these parameters is high, the level of compliance will be high and therefore, there will be less dose distribution calculation error.^8^ The advantages and disadvantages of each of the detectors (EDGE detector and EBT3 film) are important in determining the interleaf leakage, intraleaf transmission, and total average MLC leakage.

Measuring MLC leakage using very low MU, quick access to detailed results without need to process the measured data, as well as high spatial resolution and precision are the main advantages of the EDGE detector in determining the dosimetric characteristics of MLC. On the contrary, Gafchromic films have other distinctive features in this regard. These features include excellent spatial resolution and sensitivity, independence of the film's response to the energy and film reading with inexpensive equipment.^10^, ^31^ In addition, films are able to measure planar dose distributions. However, films do not have a suitable response at very low‐dose levels because in this range, the film uncertainty (errors of measurement and fitting in calibration curve) is high.^9^, ^32^ Measurement uncertainties can be due to any of the following reasons: changes in accelerator output in the time required for film exposure, natural uniformities in the sensitive layer (sensitive material called crystalline diacetylene monomer) and its thickness in different areas of the film, the possibility of statistical error of the film response at the same exposure dose levels and stochastic variations related to the readout device and conditions of film scanning. Fitting uncertainties are mainly due to the uncertainty in the process of curve fitting to the experimental data (netOD‐dose data). In addition, the other interfering factors may be involved, such as the dependence of film response to low‐energy photons (especially for low‐energy scattered photons) and changes in the dose rates.^10^, ^32^ However, by implementing a very strict protocol and attention to technical advices, film dosimetry can achieve excellent results. According to the subjects mentioned, the findings of this study indicate that the EDGE detector in comparison with the film could be an appropriate tool for measuring the dosimetric characteristics of MLCs.

As indicated in Fig. 6, the amount of interleaf leakage in leaves with a width of 3.6 mm (projected at isocenter) is more as compared with leaves with a width of 4.8 mm. In other words, the amount of leakage in the central leaves is more than that in the outer leaves because; radiation intensity is more reduced by increasing the width of the leaves. Due to the different arrangement of leaves next to each other, dose fluctuations in the leakage profile are non‐uniform. In addition, the leaf structure, design, and shape of the leaf and energy spectrum can also be affected. Therefore, these factors caused the scatter space distribution in the center of the profile to be higher than other areas and eventually, the amount of transmission became more than that of other areas of the profile.^33^

As Table 3 shows, the gamma index pass rate for Monte Carlo calculations is greater than the FSC algorithm. This is because the Monte Carlo simulation uses more accurate physical aspects (taking into account the interaction of different types of particles such as photons, electrons, and positrons with matter). More importantly, in the Monte Carlo simulation, the detailed geometric and dosimetric properties of the MLC can be properly considered. Nevertheless, several factors may result in differences between the Monte Carlo simulation and experimental measurements. The most important factors include statistical uncertainty in computer calculations carried out by Monte Carlo, mismatch in the geometry and materials of linac head and MLC, as well as systematic^34^ and random errors in water phantom measurements. Regardless of the foregoing, the Monte Carlo simulation can lead to more accurate results, if the number of particles increases and the voxel size is smaller than the scanning resolution. Of course, this would require a lot of time. However, in the current research, a good agreement was obtained between the simulated and measured dose distribution (IMRT QA field).

## 5 Conclusion

The Siemens HDP 6 MV head, together with the external LinaTech DMLC H were simulated according to the specifications of their manufacturers. The dosimetric specifications of the MLC were determined using diode detector measurements, film dosimetry data and MC simulation. BEAMnrc and DOSXYZnrc user codes were used in the commissioning of DMLC H. A good agreement was observed between the modeled and practical measured data. According to the recommendations of TG‐50, the average leaf and interleaf transmission should be less than 2% and these results demonstrated that the leakage characteristics of DMLC H satisfied international standards.

To evaluate the accuracy of the MC simulation, especially in the case of the modeling of the DMLC H, the dose distribution of the simulated IMRT field was compared with the EBT3 film dose distribution. The gamma analysis of IMRT QA showed that there is an acceptable agreement MC simulation and the experimental data. Furthermore, it was observed that the FSC algorithm has a suitable capability in dose calculation for IMRT treatments.

## Conflict of interest

The authors declare that they have no conflicts of interest.
