# Correction to “Dosimetric characteristics of LinaTech DMLC H multi leaf collimator: Monte Carlo simulation and experimental study”

**DOI:** 10.1002/acm2.70155

**Published:** 2025-07-25

Molazadeh M, Zeinali A, Robatjazi M, Shirazi A, Geraily G. Dosimetric characteristics of LinaTech DMLC H multi leaf collimator: Monte Carlo simulation and experimental study. *J Appl Clin Med Phys*. 2017;18(2):113‐124.

In paragraph 1 of the “ACKNOWLEDGMENTS” section, the text “This research was supported by the vice‐chancellor of research at Tehran University of Medical Sciences and Health Services (grant number 28202).” was incorrect and the code of ethics has been forgotten. This should have read: “This research was supported by the vice‐chancellor of research at Tehran University of Medical Sciences and Health Services (grant number 28202, Ethic code: IR.TUMS.REC.1394.164).” The online version of this article has been corrected accordingly.

We apologize for this error.

